# A new infinite family of star normal quotient graphs of twisted wreath type

**DOI:** 10.1007/s10801-022-01189-0

**Published:** 2022-12-29

**Authors:** Eda Kaja, Luke Morgan

**Affiliations:** 1 TU Darmstadt, S2|15 217, Schlossgartenstr. 7, 64289 Darmstadt, Germany; 2 University of Primorska, UP FAMNIT, Glagoljaška 8, 6000 Koper, Slovenia; 3 University of Primorska, UP IAM, Muzejski trg 2, 6000 Koper, Slovenia

**Keywords:** Group, Graph, Locally 2-arc transitive graphs, Quasiprimitive groups, Twisted wreath products, Primary 20B25, Secondary 05E18

## Abstract

We construct the first infinite families of locally 2-arc transitive graphs with the property that the automorphism group has two orbits on vertices and is quasiprimitive on exactly one orbit, of twisted wreath type. This work contributes to Giudici, Li and Praeger’s program for the classification of locally 2-arc transitive graphs by showing that the star normal quotient twisted wreath category also contains infinitely many graphs.

## Introduction

Let $\Gamma$ be a connected, finite, simple graph with vertex set $V\Gamma$, edge set $E\Gamma$ and automorphism group $\mathop {\textrm{Aut}}(\Gamma )$. An *s*-*arc* in $\Gamma$ is a tuple $(v_0,v_1,\ldots , v_s)$ such that $v_i$ is adjacent to $v_{i+1}$ and $v_{i-1}\ne v_{i+1}$ for all *i*. For $G\leqslant \mathop {\textrm{Aut}}(\Gamma )$ the graph $\Gamma$ is *locally *(*G*, *s*)*-arc transitive* if $\Gamma$ contains an *s*-arc and if for each $v \in V\Gamma$ the stabiliser of *v* in *G* acts transitively on the set of *s*-arcs emanating from *v*. If *G* is transitive on $V\Gamma$, then we say $\Gamma$ is (*G*, *s*)-*arc transitive*. We also say that $\Gamma$ is (locally) *s*-arc transitive if it is (locally) $(\mathop {\textrm{Aut}}(\Gamma ),s)$-arc transitive. If there is a permutation group *P* such that for each $v\in V\Gamma$ the permutation group induced on the neighbourhood of *v* by $G_v$ is equal to *P*, we say that *G* is *locally* *P*. It is natural to expect that (locally) *s*-arc transitive graphs with large values of *s* are interesting. Weiss [30] showed (building on work of Tutte [27, 28] in the cubic case) that $s \leqslant 7$ for all *s*-arc transitive graphs of valency at least three. Recently, van Bon and Stellmacher [29] have shown that $s\leqslant 9$ for all locally *s*-arc transitive graphs of valency at least three. Based on these results, one might hope to classify such graphs, for particular values of *s*, or with particular properties. In the vertex-transitive case, this problem is well studied; in this article we focus on the vertex-intransitive case.

Giudici et al. [7] initiated a program of global analysis aimed towards characterising locally (*G*, *s*)-arc transitive graphs with $s\geqslant 2$ such that *G* is vertex-intransitive. Such graphs are bipartite and the two parts of the bipartition are *G*-orbits. One of the main aspects of their program is a reduction [7, Theorem 1.1] to the case where *G* acts quasiprimitively on at least one of the two orbits, a so-called ‘basic’ graph. Further analysis of the basic examples can be done by utilising Praeger’s structure theorem for quasiprimitive groups [20], which divides the quasiprimitive groups into eight types—holomorph affine (HA), almost simple (AS), simple diagonal (SD), compound diagonal (CD), holomorph simple (HS), holomorph compound (HC), product action (PA) and twisted wreath (TW) (see Sect. 2 below for details). When *G* is quasiprimitive on both orbits [7, Theorem 1.2] shows that either the quasiprimitive type on each orbit is the same or is a pairing of SD and PA types. All the examples are known in the latter case (see [11]), and in the former case a classification of such graphs would imply a classification of basic (*G*, *s*)-arc transitive graphs (see the discussion preceding [7, Lemma 3.3]) and is therefore incomplete. When *G* is quasiprimitive on exactly one orbit, [7, Theorem 1.3] shows that the quasiprimitive type is HA, HS, AS, PA or TW. A full classification was achieved in the HA and HS cases (see [8, Theorem 1.4] and [9, Theorem 1.1]), and in the AS case the possible simple groups that can appear is determined [9, Theorem 1.3]. In the PA case, much is known if the action is primitive rather than quasiprimitive (see [9, Theorem 1.2]), and an infinite family of such graphs is given. In the TW case however, only one example was provided.

The twisted wreath product construction was introduced by Neumann [18] and refined by Suzuki [25]. Not every group that arises from the construction is primitive or even quasiprimitive—the precise conditions are delicate; in fact the twisted wreath groups were missed in the first version of the O’Nan–Scott theorem for primitive groups [24]. We refer the reader to Baddeley [2] for a discussion of the conditions for which a group of twisted wreath type is primitive. Furthermore, Baddeley [3] has characterised those 2-arc transitive graphs with automorphism group a quasiprimitive group of TW type.

In this paper, we construct infinitely many locally (*G*, 2)-arc transitive graphs, where *G* is vertex-intransitive and is quasiprimitive on exactly one orbit of TW type. Our main result thus shows that the last category of Giudici, Li and Praeger’s global analysis program described above is also infinitely populated. Using results on inclusions of quasiprimitive groups [4, 22], we show that the automorphism group of our constructed graphs has the same properties as *G*, and that their *socles* (the product of all their minimal normal subgroups) are equal, so that the groups are closely related. This also proves that the graphs are basic and that the graphs we construct are *new*, in the sense that they do not arise from one of the constructions in [7], or the sequels [9–11] or further studies of locally *s*-arc transitive graphs [6, 8, 12, 16, 26].

### Theorem 1.1

For each 2-transitive group *P* of degree *k* appearing in Table 1 there exists a graph $\Gamma$ of valency *k* and a group $G\leqslant \mathop {\textrm{Aut}}(\Gamma )$ such that $\Gamma$ is locally (*G*, 2)-arc transitive and *G* is locally *P*. Moreover, the following hold for $A = \textrm{Aut}(\Gamma )$:

1. *A* has two orbits $\Delta _1$ and $\Delta _2$ on $V\Gamma$;
2. the action of *G* and the action of *A* on $\Delta _1$ are quasiprimitive of twisted wreath type;
3. $\textrm{soc}(G)=\textrm{soc}(A)$ and $\textrm{soc}(A)$ is intransitive on $\Delta _2$ with quotient graph $\Gamma _{\textrm{soc}(A)}$ the complete bipartite graph $K_{1,k }$.

In part (3) of the theorem, the vertex set of the *quotient graph* $\Gamma _{\textrm{soc}(A)}$ is the set of $\textrm{soc}(A)$-orbits in $V\Gamma$ and there is an edge in $\Gamma _Y$ between two vertices if there is an edge between the corresponding $\textrm{soc}(A)$-orbits in $\Gamma$. A complete bipartite graph $K_{1,k}$ is usually called a *star*. It is these properties that give rise to the name ‘star normal quotient’ to describe graphs such as those appearing in Theorem 1.1.

In Sect. 2 we assemble some results on overgroups of primitive and quasiprimitive groups. The construction of the graphs appearing in the theorem takes place in Sect. 3, and in Sect. 4 we analyse the automorphism groups of the constructed graphs.

## Preliminaries

The construction of the graphs appearing in Theorem 1.1 is based on the *coset graph* construction.

### Definition 2.1

Let *G* be a group and let $L,R < G$ be such that $L\cap R$ is core-free in *G*. Let $\Delta _1=\{Lx:x\in G\}$ and let $\Delta _2=\{Ry:y\in G\}$. Define the bipartite graph $\Gamma =\textrm{Cos}(G,L,R)$ such that $V\Gamma =\Delta _1\cup \Delta _2$ and $Lx\sim Ry$ if and only if $Lx \cap Ry \ne \emptyset$. We refer to $(L,R,L\cap R)$ as the associated *amalgam*.

The following lemma shows that every locally *s*-arc transitive graph arises from the above construction.

### Lemma 2.2

[7, Lemma 3.7] For a group *G* and subgroups $L,R < G$ such that $L\cap R$ is core-free in *G*, the graph $\Gamma =\textrm{Cos}(G,L,R)$ satisfies the following properties:

1. $\Gamma$ is connected if and only if $\langle L,R \rangle = G$;
2. $G\leqslant \textrm{Aut}(\Gamma )$ and $\Gamma$ is *G*-edge transitive and *G*-vertex intransitive;
3. *G* acts faithfully on both $\Delta _1$ and $\Delta _2$ if and only if both *L* and *R* are core-free.

Conversely, if $\Gamma$ is *G*-edge transitive and not *G*-vertex transitive, and *v* and *w* are adjacent vertices, then $\Gamma \cong \textrm{Cos}(G,G_v,G_w)$.

A transitive permutation group *G* on a finite set $\Omega$ is *quasiprimitive* if each non-trivial normal subgroup of *G* acts transitively on $\Omega$. Praeger [20] classified quasiprimitive groups in an O’Nan–Scott type theorem and separated them into eight types [21] (see also [7, Section 2]). The classification is based around the abstract structure and the action of the *socle* (the product of the minimal normal subgroups). If *G* is a finite quasiprimitive permutation group with socle *X*, then either *X* itself is a minimal normal subgroup, or $X=X_1X_2$ for some minimal normal subgroups $X_1$ and $X_2$ of *G*. In the latter case, $X_1 \cong X_2 \cong T^k$ for some finite non-abelian simple group *T* and integer *k*; if $k=1$ then *G* is of holomorph simple (HS) type and if $k>1$ then *G* is of holomorph compound (HC) type. If *X* itself is a minimal normal subgroup of *G*, then either *X* is abelian and regular and *G* is of holomorph affine (HA) type, or, $X\cong T^k$ for some non-abelian simple group *T*. If $k=1$ then *G* is of almost simple (AS) type. For $k>1$, the type depends upon the structure of a point stabiliser, *H* say, in *X*. If $H=1$ then *X* is regular and *G* is of twisted wreath (TW) type. If *H* is non-trivial and projects to a proper subgroup in each of the simple direct factors of *X*, then *G* is of product action (PA) type. If *H* is non-trivial and projects onto each of the simple direct factors, then *G* is of simple diagonal (SD) type if $H\cong T$ and of compound diagonal (CD) type if $H \cong T^\ell$ for some integer $\ell >1$.

We will require the following results about the inclusion problem of primitive and quasiprimitive groups. Recall that a permutation group *G* acting on a set $\Omega$ is primitive if the only partitions of $\Omega$ that are preserved by *G* are the partition of $\Omega$ into singletons and the partition $\{ \Omega \}$. For any $\omega \in \Omega$, there is a one-to-one correspondence between the overgroups of the point stabiliser $G_\omega$ of $\omega$ in *G* and the partitions of $\Omega$ that are preserved by *G*. Thus a transitive permutation group is primitive if and only if point stabilisers are maximal subgroups.

### Lemma 2.3

[19, Proposition 8.1] Suppose that $G \leqslant H \leqslant \mathop {\textrm{Sym}}(n)$ and that *G* is primitive. If *G* has type SD, then one of the following holds: $H=\mathop {\textrm{Alt}}(n)$; $H=\mathop {\textrm{Sym}}(n)$; $\textrm{soc}(G)=\textrm{soc}(H)$ and *H* is of SD type.

### Lemma 2.4

Suppose that $G \leqslant H \leqslant \mathop {\textrm{Sym}}(n)$ and that *G* is quasiprimitive. If *G* has type TW and if *H* is primitive, then one of the following holds:

1. $$H=\mathop {\textrm{Alt}}(n)$$ or $$\mathop {\textrm{Sym}}(n)$$;
2. *H* is of type PA, HC, SD or CD;
3. *H* is of type TW and $\textrm{soc}(G)=\textrm{soc}(H)$.

### Proof

This follows from [4, Theorem 1.2]. $\square$

If *G* is a permutation group acting on a set $\Omega$ and *G* preserves a partition ${\mathcal {B}}$ of $\Omega$, then $G^{\mathcal B}$ denotes the permutation group induced on ${\mathcal {B}}$ by *G*.

### Lemma 2.5

Suppose that $G \leqslant H \leqslant \mathop {\textrm{Sym}}(n)$ and that *G* is quasiprimitive. If *G* has type TW and *H* is imprimitive, then for a partition ${\mathcal {B}}$ that is preserved by *H*, one of the following holds:

1. *H* has type TW and $\textrm{soc}(G)=\textrm{soc}(H)$,
2. $\textrm{soc}(G)=T^k < \textrm{soc}(H) = S^k$ for non-abelian simple groups *S*, *T*, and both $G^{{\mathcal {B}}}$ and $H^{{\mathcal {B}}}$ are of type PA.

### Proof

This follows from [22, Theorem 2]. $\square$

If *G* is a transitive permutation group acting on the set $\Omega$ and *Y* is an intransitive normal subgroup of *G*, then the set of orbits of *Y* on $\Omega$, denoted by $\Omega /Y$, forms a partition of $\Omega$ that is preserved by *G*.

## A construction

We assume the following hypothesis throughout this section.

### Hypothesis 3.1

1. $P = V \rtimes Q$ is an affine 2-transitive group of degree *k*;
2. the stabiliser $Q_x$ of a nonzero vector $x \in V$ has a non-trivial centre with order divisible by the unique prime *p* that divides *k*;
3. there is a non-abelian finite simple group *T* and a homomorphism $\phi : Q \rightarrow \mathop {\textrm{Aut}}(T)$ such that $\phi (Q)$ contains $\textrm{Inn}(T)$.

The groups that satisfy (1) were classified by Huppert [15] and Hering [14] (see also [17, Appendix 1]). From this family, we find that the groups listed in Table 1 satisfy Hypothesis 3.1. In the table below, *m* is an integer and by $E_{q^2}$ denotes an elementary abelian group of order $q^2$.

**Table 1:** Ingredients of the group $G=T\;, \textrm{twr}_\phi P$

*k*	*P*	*Q*	$Q_x$	$|Z(Q_x)|$	*T*	Notes
$q^2$	$q^2 : \mathop {\textrm{SL}}(2,q)$	$\mathop {\textrm{SL}}(2,q)$	$E_{q^2}$	*q*	$\mathop {\textrm{PSL}}(2,q)$	$q>3$
$q^{2m}$	$q^{2m} : \mathop {\textrm{Sp}}(2m,q)$	$\mathop {\textrm{Sp}}(2m,q)$	$q.q^{2(m-1)}.\mathop {\textrm{Sp}}(2(m-1),q)$	*q*	$\textrm{PSp}(2m,q)$	
$3^4$	$3^4 : \mathop {\textrm{SL}}(2,5).2$	$\mathop {\textrm{SL}}(2,5).2$	$C_3$	3	$\mathop {\textrm{PSL}}(2,5)$	
$3^6$	$3^6 : \mathop {\textrm{SL}}(2,13)$	$\mathop {\textrm{SL}}(2,13)$	$C_3$	3	$\mathop {\textrm{PSL}}(2,13)$	

Define

3.1 $$\begin{aligned} G = T\, \textrm{twr}_\phi P, \end{aligned}$$

and identify *P* and *Q* as subgroups of *G*. Let *N* be the base group of *G* so that

$$\begin{aligned} N = \{ f : P\rightarrow T \mid f(hx) = f(h)^{\phi (x)}\text { for all } h\in P, \, x\in Q \}. \end{aligned}$$

For $g\in P$, the action of *g* on *N* is

$$\begin{aligned} f^g (h) = f (gh), \quad h\in P. \end{aligned}$$

Fix a left transversal $z_1,\ldots ,z_k$ to *Q* in *P* so that each $f\in N$ is uniquely determined by the images $f(z_1)$, $f(z_2),\ldots , f(z_k)$. In this way, we have $N \cong T^k$. For $1\leqslant i \leqslant n$ we define

3.2 $$\begin{aligned} T_i = \{ f\in N \mid f(z_j) = 1 \text { for } j\ne i \} \quad \text {and} \quad N_i = \{ f\in N \mid f(z_i) = 1\}. \end{aligned}$$

The action of *G* by conjugation on the set $\{T_1,\ldots ,T_k\}$ is permutationally equivalent to the action of *P* on the coset space [*P* : *Q*]. With respect to this, we have $Q= N_P(T_k) = N_P( N_k)$.

Since *V* is a complement to *Q* in *P*, we may take the elements of *V* to be a left transversal of *Q* in *P*. We record the following observation: if $x\in Q$ is such that $xz_i = z_i x$, then for $f\in T_i$ we have

$$\begin{aligned} f^x(z_i) = f(xz_i) = f(z_ix) = f(z_i)^{\phi (x)} \end{aligned}$$

so that *x* induces on $T_i\cong T$ the automorphism $\phi (x)$.

We view *G* as a permutation group on the set [*G* : *P*]. Since $\textrm{core}_P(Q) = 1$, *G* acts faithfully on [*G* : *P*] and *G* is a quasiprimitive group of twisted wreath type (see [20, Section 2]).

### Lemma 3.2

The group *G*, as a permutation group on the set [*G* : *P*], is quasiprimitive of TW type. The action is imprimitive, and *G* preserves a unique (non-trivial) partition $\Pi$ which corresponds to the overgroup $PC_N( V \ker (\phi ))$ of *P*. The induced action of *G* on $\Pi$ is primitive of type SD and for $\pi \in \Pi$ the action of $G_\pi$ on $\pi$ is primitive of type HS.

### Proof

First we gain some insight on the possible partitions preserved by *G*. Since $G=NP$, if $P \leqslant H < G$, then $H = P ( H \cap N)$. Set $M=H \cap N$ and note that *M* is normalised by *P*. Since *P* is transitive on the *k* simple direct factors of *N*, the projections of *M* to the simple direct factors are isomorphic. Further, since the projection of *M* to $T_k$ is normalised by *Q* and *Q* induces $\textrm{Inn}(T_k)$ on $T_k$ by conjugation, each projection is either trivial (and hence $M=1$) or each projection is surjective.

It follows that *M* is a subdirect subgroup of *N*, and therefore by Scott’s lemma (see [23, Theorem 4.16]) that *M* is a product of strips, that is, $M= \prod _{i\in I} D_i$ where *I* forms a partition of $\{1,\ldots ,k\}$ and each $D_i$ is a diagonal subgroup of $\prod _{ j\in I_i} T_j$ where $I = I_1 \cup \ldots \cup I_{|I|}$. Clearly the set *I* forms a partition preserved by *G*. Since *G* is primitive on the set $\{T_1,\dots ,T_k\}$ of simple direct factors of *N*, *I* is a trivial partition. Since *M* is a proper subgroup of *N*, we have $|I|=1$ and thus *M* is a full diagonal subgroup of *N*. Hence $M\cong T$. Since *P* normalises *M*, we have a homomorphism $c : P \rightarrow \mathop {\textrm{Aut}}(T)$ which is the map induced by conjugation. Since *Q* induces $\textrm{Inn}(T)$ on $T_k\cong T$ we see that $c(Q) = \textrm{Inn}(M)$. This means $\mathop {\textrm{Inn}}(M)$ normalises *c*(*V*), and since *c*(*V*) is elementary abelian, the only possibility is that $c(V)=1$. Hence $V \leqslant C_P(M)$ and $C_P(M) = V C_Q(M)$. Again, since *Q* must induce $\textrm{Inn}(T)$ on $M \cong T$, we have that $C_Q(M)=\ker (\phi )$ so that $C_P(M)=V \ker (\phi )$ and $M \leqslant C_N(V \ker (\phi ))$.

Thus we have proved that the only overgroups of *P* correspond to subgroups of $C_N(V\ker (\phi ))$ that are normalised by *Q*. We proceed to analyse the action of *Q* on $C_N(V\ker (\phi ))$ to find all partitions that are preserved by *G*.

Since *Q* is maximal in *P* and $P=VQ$, the map $\phi :Q \rightarrow \mathop {\textrm{Aut}}(T)$ can be extended (uniquely) to the whole of *P*, and this extension is a homomorphism ${\hat{\phi }}$ with kernel $V\ker (\phi )$. The twisted wreath product $T \textrm{twr}_{{\hat{\phi }}} P$ shows that *G* is imprimitive, since the subgroup

3.3 $$\begin{aligned} {\hat{N}} = \{ f : P \rightarrow T \mid f(gh ) = f(g)^{{\hat{\phi }}(h)} \text { for all } g,h\in P \} \end{aligned}$$

is a subgroup of *N* that is normalised by *P*. Further, ${\hat{N}} \cong T^\ell$ where $\ell =|P:P|=1$. Let $f\in C_N(V\ker (\phi ))$ and let $g,h\in P$. Write $g=ab$ and $h = cd$ for $a,c\in V$ and $b,d\in Q$. Using the fact that *V* is abelian and that $c^{b^{-1}} \in V$, we find

$$\begin{aligned} f(gh)&= f(abcd) = f(abc)^{\phi (d)} = f(abc)^{{\hat{\phi }}(cd)} = f(c^{b^{-1}} ab)^{{\hat{\phi }}(h)}\\&=f^{c^{b^{-1}}}(ab)^{{\hat{\phi }}(h)} =f(g)^{{\hat{\phi }}(h)} \end{aligned}$$

and so $f \in {\hat{N}}$. Thus $C_N(V\ker (\phi )) \leqslant {\hat{N}}.$ The subgroup ${\hat{N}}$ can be identified as the “first-coordinate” subgroup of the twisted wreath product $T \textrm{twr}_{{\hat{\phi }}} P$, so that ${\hat{N}} = \{ f_t : t\in T\}$ where $f_t : P \rightarrow T$ is defined by

$$\begin{aligned} f_t (h) = t^{{\hat{\phi }}(h)} \end{aligned}$$

and in this way, for any $r \in V\ker (\phi )$ and for any $h\in P$ we have

$$\begin{aligned} (f_t)^r (h) = f_t(rh) = t^{{\hat{\phi }}(rh)} = t^{{\hat{\phi }}(h)} = f_t(h) \end{aligned}$$

so that $V\ker (\phi )$ centralises ${\hat{N}}$. Hence ${\hat{N}} = C_N( V\ker (\phi ) )$. If $x \in Q$ and $t\in T$ we see that

$$\begin{aligned} (f_t)^x (1) = f_t(x) = t^{{\hat{\phi }}(x)}=t^{\phi (x)} = f_{t^{\phi (x)}}(1) \end{aligned}$$

so that *Q* induces $\mathop {\textrm{Inn}}(T)$ on ${\hat{N}}\cong T$. In particular, this shows that *Q* normalises no proper non-trivial subgroup of $C_N(V\ker (\phi ))$, and so the only possible overgroups of *P* in *G* are $C_N(V\ker (\phi ))P$ and *G* itself.

Let $\Pi$ denote the partition that is given by the overgroup ${\hat{N}} P$ and let $\pi$ denote the orbit of ${\hat{N}}$ so that $G_\pi ={\hat{N}}P$. Since *G* is quasiprimitive, the action of *G* on $\Pi$ is faithful and quasiprimitive. Further, since $N_\pi = {\hat{N}}$ is a full diagonal subgroup of *N*, the action is of SD type. The action of *G* on the set of simple direct factors of *N* is equivalent to the action of *P* on the set of vectors of *V* and is therefore primitive. Hence $G^{\Pi }$ is a primitive group of SD type.

Finally, consider the action of $G_\pi = {\hat{N}}P$ on $\pi$. The kernel of the action is the largest subgroup of *P* normalised by ${\hat{N}}$, and such a subgroup must commute with ${\hat{N}}$. From above, we have that the kernel of the action is $V\ker (\phi )$. Thus

$$\begin{aligned} G_\pi ^\pi = {\hat{N}} \rtimes P / (V \ker (\phi )) = {\hat{N}} \rtimes \phi (Q) \end{aligned}$$

and since $N_\pi ^\pi \cong T$ is a normal subgroup, $G_\pi ^\pi$ is primitive of type HS. $\square$

Recall the definition of $N_k$ from (3.2).

### Lemma 3.3

There exists a subgroup *R* of $N_{k}$ of order |*V*| that is normalised by *Q*, and $RQ \cong P$.

### Proof

Recall that $Q=N_P(T_k)=N_P(N_k)$ and for $i=1,\ldots ,k-1$, set $Q_i = N_Q(T_i)$. By Hypothesis 3.1, the centre of $Q_i$ is non-trivial and has order divisible by *p*. Further, $Q_1$ induces inner automorphisms on $T_1$ corresponding to the image of $Q_1$ under the map $\phi$ and therefore normalises a subgroup $U_1$ of order *p*. For $g\in Q$ such that $(T_1)^g=T_i$, let $U_i = (U_1)^g$ (and note that the definition of $U_i$ is independent of the choice of *g* since $Q_1=N_Q(T_1)$). Let $W = \langle U_1,\ldots ,U_{k-1} \rangle$. Since $U_i \leqslant T_i$, we have $[U_i,U_j]=1$ for all *i*, *j*. Thus *W* is an elementary abelian group of order $p^{k-1}$ and *W* is normalised by *Q*. Further, since $U_1$ is the trivial module for $Q_1$, *W* is the permutation module for *Q* of dimension $k-1$ and there is a basis for *W* which *Q* permutes as it does the set of nonzero vectors of *V*.

(Aside: to see this directly, pick $u_1 \in U_1$ so that $U_1 = \langle u_1\rangle$. Then set $u_i = (u_1)^g$ if $g\in Q$ is such that $(T_1)^g = T_i$. Let $z_1,\ldots ,z_{k-1}$ be a right transversal to $Q_1$ in *Q*, then for $g\in Q$ there is $r\in Q_1$ and *i* such that $g=rz_i$, then

$$\begin{aligned} (u_1)^g = (u_1)^{rz_i} = u_1^{z_i} = u_i \end{aligned}$$

and hence for any *j*, there is $h\in Q$ such that

$$\begin{aligned} (u_j)^g = ((u_1)^h)^g = (u_1)^{hg} = u_s \end{aligned}$$

for some *s*. Hence *Q* simply permutes the elements $\{u_1,\ldots ,u_{k-1}\}$ which form a generating set for *W*, and therefore *W* is simply the permutation module for *Q* with a basis corresponding to the nonzero vectors of *V*.)

Since $Q_1$ fixes the vector $x \in V$, it preserves the 1-dimensional subspace $\langle x \rangle$ of *V* that is isomorphic to $U_1$. Thus there is an injective $Q_1$-module homomorphism $f: U_1 \rightarrow V$. By [1, Frobenius Reciprocity Theorem, pg. 165], there is a *Q*-module homomorphism $F: W \rightarrow V$ such that *F* extends *f*. In particular, *F* is also nonzero, and therefore *F* is surjective since *V* is an irreducible *Q*-module. Thus there is a submodule *Y* of *W* such that $W/Y \cong V$. Now *W* is self-dual since the permutation module supports a *Q*-invariant symmetric bilinear form. Hence there is a submodule, *R* say, of *W* such that $R \cong V$ as a *Q*-module. Hence *Q* acts on *R* as it does on *V*, and $RQ \cong P$. $\square$

### Remark 3.4

Although Hypothesis 3.1 does not hold for $P= \mathop {\textrm{ASL}}(3,2) \cong 2^3 \rtimes \mathop {\textrm{GL}}(3,2)=VQ$—for a nonzero vector *x* we have $Q_x \cong 2^2 : \mathop {\textrm{SL}}(2,2)$, so that $Q_x$ has trivial centre—if *W* is the $\textrm{GF}(2)Q$-module of dimension 14 induced from the natural module of $Q_x/O_2(Q_x) = \mathop {\textrm{SL}}(2,2)$, then computations in Magma [5] show that there is a submodule of *W* of dimension 3. Whilst the same conclusion holds for $P=\mathop {\textrm{ASL}}(4,2)$, it fails to hold for $P=\mathop {\textrm{ASL}}(5,2)$.

We define the following coset graph

3.4 $$\begin{aligned} \Gamma = \textrm{Cos}(G,P,RQ) \end{aligned}$$

with *R* as in Lemma 3.3. Let *u* and *v* denote the cosets *P* and *RQ*, respectively, so that $G_u = P$ and $G_v = RQ$. Let $\Delta _1=u^G$ and $\Delta _2 = v^G$.

### Lemma 3.5

The graph $\Gamma$ possesses the following properties:

1. $\Gamma$ is connected and of valency $k=|V|$;
2. $\Gamma$ is locally (*G*, 2)-arc transitive and *G* is locally *P*;
3. *G* acts faithfully on the two orbits, $\Delta _1$ and $\Delta _2$;
4. $G^{\Delta _1}$ is quasiprimitive of TW type;
5. $G^{\Delta _2}$ is not quasiprimitive; *N* is intransitive with *k* orbits.

### Proof

By Lemma 3.2, $PC_N(V \ker (\phi ))$ is the unique overgroup of *P* in *G*, and since *Q* induces the inner automorphism group on $C_N(V \ker (\phi )))\cong T$, we have that $R \nleqslant C_N(V \ker (\phi ))$. Thus $G = \langle P, RQ \rangle$ and $\Gamma$ is connected. From the maximality of *Q* in *P* and in *RQ*, we have $Q = P \cap RQ$. It follows that $\Gamma$ has valency *k*. The actions of *P* and *RQ* on the sets [*P* : *Q*] and [*RQ* : *Q*] are both equivalent to the 2-transitive action of *P* on *k* points, and hence, $\Gamma$ is locally (*G*, 2)-arc transitive and *G* is locally *P*. Clearly *P* and *RQ* have trivial core in *G*, and therefore *G* is faithful on both $\Delta _1$ and $\Delta _2$. The action of *G* on $\Delta _1$ is as the TW group first constructed above. Since $RQ \leqslant NQ < G$, the action of *G* on $\Delta _2$ is not quasiprimitive, and *N* has $|G:NQ|=|P:Q|=k$ orbits on $\Delta _2$. $\square$

## The automorphism group of $\Gamma$

We continue with the notation from the previous section, so that *G* is the group defined in (3.1), $\Gamma$ is the graph defined in (3.4) and $\Pi$ and $\pi$ are as in Lemma 3.2. Since $\Gamma$ is bipartite, we let *A* be the normal subgroup of $\mathop {\textrm{Aut}}(\Gamma )$ that preserves the bipartition which has index at most two in $\mathop {\textrm{Aut}}(\Gamma )$. We will establish that the actions of *A* on the two parts of $\Gamma$ are not equivalent, and this will show that $A=\mathop {\textrm{Aut}}(\Gamma )$. Let $s\in {\mathbb {N}}$ be such that *A* is locally *s*-transitive. Since $\Gamma$ is locally (*G*, 2)-arc transitive, we have that $s\geqslant 2$.

### Lemma 4.1

If $1\ne X$ is a normal subgroup of *A* that is intransitive on $\Delta _1$, then the set of orbits of *X* on $\Delta _1$ is $\Pi$ and $A^\Pi$ is primitive. Further, if *X* is also intransitive on $\Delta _2$, then $A^\Pi$ does not contain $\mathop {\textrm{Alt}}(\Pi )$.

### Proof

Lemma 3.2 shows that $\Pi$ is the unique partition of $\Delta _1$ that is preserved by *G*; hence, the set of orbits of *X* is $\Pi$ and $A^\Pi$ is primitive. Now assume that *X* is intransitive on $\Delta _2$. By [7, Lemma 5.1], we have that *X* is semiregular on both $\Delta _1$ and $\Delta _2$. Further, we have $|X|=|\pi |$ and *X* is a normal subgroup of $J= \langle X, {\hat{N}} , P\rangle$, where ${\hat{N}}$ is the group defined in (3.3). By Lemma 3.2${\hat{N}} P=G_\pi$ acts on $\pi$ as a primitive group of HS type, so *J* is also primitive on $\pi$ and [19, Proposition 8.1] shows that $J^\pi$ is $\mathop {\textrm{Alt}}(\pi )$, $\mathop {\textrm{Sym}}(\pi )$, has type SD or type HS. Of the possibilities, only HS groups have normal subgroups of order $|X|=|T|$, so therefore $J^\pi$ is primitive of type HS and we have $X \cong T$.

Suppose, for a contradiction, that $A/X = A^{\Pi }$ contains $\mathop {\textrm{Alt}}(\Pi )$. We claim that $C_A(X)$ contains a normal subgroup isomorphic to $\mathop {\textrm{Alt}}(\Pi )$. Since $C_A(X) \cap X = Z(X)=1$, we have that $C_A(X) \cong C_A(X) X / X \unlhd A/X$. Hence the claim is true, unless $C_A(X)=1$. In this case, then *A* embeds into $\mathop {\textrm{Aut}}(T)$. The order of $\mathop {\textrm{Aut}}(T)$ divides |*T*|!, and yet $|\mathop {\textrm{Alt}}(\Pi )|=(|T|^{k-1})!/2$ divides |*A*|, which is a contradiction since $k>2$. Hence $C_A(X)$ contains a normal subgroup of index at most two that is isomorphic to $\mathop {\textrm{Alt}}(\Pi )$, thus $[C_A(X),C_A(X)]\cong \mathop {\textrm{Alt}}(\Pi )$. Let $H=X [C_A(X),C_A(X)]$ and note that $|A:H| \leqslant 2$. Hence $|G : G\cap H| \leqslant 2$. Thus $|G_v : G_v \cap H| \leqslant 2$ and $|G_u : G_u \cap H| \leqslant 2$. In particular, both $G_v \cap H_v = G_v \cap H$ and $G_u \cap H_u=G_u \cap H$ are normal subgroups of $G_v$ and $G_u$, respectively. Since the valency of $\Gamma$ is at least three, and since $G_v$ and $G_u$ act 2-transitively on $\Gamma (v)$ and $\Gamma (u)$, respectively, it must be that $G_v \cap H$ and $G_u \cap H$ are transitive on $\Gamma (v)$ and $\Gamma (u)$, respectively. Hence *H* is locally transitive, so $H = \langle H_u, H_v \rangle$. Order considerations yield:

$$\begin{aligned} |H| = |X| | \mathop {\textrm{Alt}}(\Pi )| = |T| \frac{(|T|^{p^2-1})!}{2} \end{aligned}$$

and

$$\begin{aligned}{} & {} |H_u| = |H_v | = \frac{|H|}{|T|^{p^2}} = \frac{|T| (|T|^{p^2-1})!}{2|T|^{p^2}}\\ {}{} & {} = \frac{ (|T|^{p^2-1})!}{2|T|^{p^2-1}} = \frac{(|T|^{p^2-1}-1)!}{2} = |\mathop {\textrm{Alt}}(|\Pi |-1)|. \end{aligned}$$

Now $H_v \cap X=1=H_u \cap X$ since *X* is semiregular on both $\Delta _1$ and $\Delta _2$, so we have that both $H_v$ and $H_u$ are isomorphic to subgroups of $\mathop {\textrm{Alt}}(\Pi )$ with the same order as a point stabiliser in $\mathop {\textrm{Alt}}(\Pi )$. It follows that $H_v \cong H_u \cong \mathop {\textrm{Alt}}(|\Pi |-1)$. In particular, both $H_v$ and $H_u$ are simple. If $H_v \cap C_H(X)$ is trivial, then $H_v$ is isomorphic to a subgroup of $H/C_H(X) \cong T$, which, as mentioned above, is impossible. Hence $H_v \leqslant C_H(X)$. Similarly $H_u \leqslant C_H(X)$ and therefore $H \leqslant C_H(X)$, which means $H = C_H(X)$. This gives $X = X \cap H = X \cap C_H(X) = Z(X) = 1$, a contradiction. $\square$

### Lemma 4.2

The identity subgroup is the only normal subgroup of *A* that is intransitive on both $\Delta _1$ and $\Delta _2$.

### Proof

Assume for a contradiction that there is a non-trivial normal subgroup *X* of *A* that is intransitive on $\Delta _1$ and $\Delta _2$. Further, after replacing *X* with an overgroup, we may assume that *X* is maximal with this property. Hence we may apply [7, Theorem 1] and consider the outcomes (i)–(iii) separately.

In the case (i), we have $\Gamma _X = K_{k,k}$. Since *G* acts quasiprimitively on $\Delta _1$, *G* is isomorphic to its image in $\mathop {\textrm{Aut}}(\Gamma _X)$. This implies that $|T|^{k}$ divides $|\mathop {\textrm{Sym}}(k)|$, and a contradiction is obtained by considering the power of *p* that divides each group.

In the case (ii), we have that $A^\Pi$ is an overgroup of a primitive group of SD type. By Lemma 4.1$A^\Pi$ does not contain the alternating group on $\Pi$. Hence by Lemma 2.3$A^\Pi$ must be of type SD. By [7, Theorem 1.2] it must be that $A^{\Delta _2/X}$ is of type PA. According to [11, Theorem 1.2], $\Gamma _X$ arises from either [11, Construction 3.3] or from a normal quotient of such a graph as in [11, Construction 3.10]. Since all such graphs are not regular, we have a contradiction.

In case (iii), *A* is quasiprimitive on exactly one of $\Delta _1/X = \Pi$ or $\Delta _2/X$. Since *G* is primitive on $\Pi$, $A^\Pi$ is primitive (and therefore quasiprimitive) on $\Pi$. Hence *A* is not quasiprimitive on $\Delta _2 / X$. By [7, Theorem 1.3], the quasiprimitive type of $A^\Pi$ is either HA, HS, AS, PA or TW. Since $G^\Pi$ is primitive of SD type, Lemma 2.3 implies that $A^\Pi$ has type AS and $A^\Pi$ contains $\mathop {\textrm{Alt}}(\Pi )$. Lemma 4.1 delivers a contradiction. $\square$

### Lemma 4.3

There is no non-trivial normal subgroup of *A* that is intransitive on $\Delta _1$. In particular, $A^{\Delta _1}$ is quasiprimitive.

### Proof

Assume for a contradiction that *Y* is a normal subgroup of *A* that is intransitive on $\Delta _1$. By the previous lemma, *Y* must be transitive on $\Delta _2$. By Lemma 3.2, the set of orbits of *Y* on $\Delta _1$ must be the set $\Pi$, and therefore $|u^Y| = |T|$. Since each vertex in $u^Y$ has *k* neighbours in $\Delta _2$, there are exactly *k*|*T*| edges between $u^Y$ and $\Delta _2$. On the other hand, $\{u^y,v^y\}$ is an edge between $u^Y$ and $\Delta _2$ for all $y\in Y$. Since *Y* is transitive on $\Delta _2$, there are at least $|\Delta _2|= |T|^k$ edges between $\Delta _2$ and $u^Y$, a contradiction. $\square$

### Lemma 4.4

The action of *A* on $\Delta _2$ is not quasiprimitive and the action of *A* on $\Delta _1$ is quasiprimitive of type TW. Moreover, $\textrm{soc}(G)=\textrm{soc}(A)$.

### Proof

Lemma 4.3 allows us to consider the inclusion $G^{\Delta _1} \leqslant A^{\Delta _1}$ of quasiprimitive groups. If $A^{\Delta _1}$ is of type TW, then Lemmas 2.4 and 2.5 show that $\textrm{soc}(G)=\textrm{soc}(A)$. Since $\textrm{soc}(G)$ is intransitive on $\Delta _2$, this shows *A* is not quasiprimitive on $\Delta _2$. For the remainder of the proof, we may therefore assume that $A^{\Delta _1}$ is not of TW type, and we aim to find a contradiction.

We consider two cases according to whether $A^{\Delta _1}$ is primitive or not.

**Case 1** $A^{\Delta _1}$ is primitive.

Since $s\geqslant 2$, $A^{\Delta _1}$ cannot be of type HC or CD by [7, Theorem 1.2] and [7, Theorem 1.3]. Hence Lemma 2.4 implies that $A^{\Delta _1}$ contains the alternating group on $\Delta _1$ or has type PA (since we assume $A^{\Delta _1}$ is not of type TW). Since $\Delta _1$ and $\Delta _2$ have the same cardinality, if $A^{\Delta _1}$ contains the alternating group, then $A^{\Delta _2}$ also contains the alternating group, and $\Gamma$ is the complete bipartite graph or the empty graph, a contradiction. Hence we may assume that $A^{\Delta _1}$ is primitive of type PA.

Suppose first that $A^{\Delta _2}$ is quasiprimitive. Since $\Gamma$ is regular, [11, Theorem 1.2] shows that the type of $A^{\Delta _2}$ must be the same type as $A^{\Delta _1}$. Hence the type of *A* (on both $\Delta _1$ and $\Delta _2$) is PA. Since $s\geqslant 2$ and since $A^{\Delta _1}$ is primitive, we obtain a contradiction from [13, Theorem 2.1].

We may now assume that $A^{\Delta _2}$ is not quasiprimitive. If $A^{\Delta _1}$ is of type PA, then [9, Theorem 1.2] shows that $\Gamma$ is the vertex-maxclique incidence graph of the Hamming graph $H(\ell ,n)$ for some integers $\ell$ and *n*. Since the vertex-maxclique incidence graph of $H(\ell ,n)$ has valencies $\ell$ and *n*, we have $\ell =n=k$. Hence the number of vertices in $\Gamma$ is $2 n^{\ell -1}\ell = 2 n^n = 2 k^{2k}$, a contradiction since *k* is a prime power.

**Case 2** $A^{\Delta _1}$ is imprimitive, and therefore preserves the partition $\Pi$. Since $G^{\Pi }$ is primitive of SD type by Lemma 3.2, then Lemma 2.3 shows $A^{\Pi }$ cannot have type PA. Hence Lemma 2.5 (1) must hold, which contradicts our assumption that $A^{\Delta _1}$ is not of TW type. $\square$

## The proof of Theorem 1.1

Let $P=V Q$ be a 2-transitive affine group that satisfies Hypothesis 3.1 (and note that this includes all the groups in Table 1). Let *G* be the twisted wreath product defined by (3.1) and let $\Gamma$ be the graph defined by (3.4). From Lemma 3.5 we have that $\Gamma$ is a connected graph of valency $k=|V|$. Let $\Delta _1$ and $\Delta _2$ denote the two parts of $\Gamma$ and let *A* be the subgroup of $\mathop {\textrm{Aut}}(\Gamma )$ fixing the two parts. Lemma 4.4 shows that *A* is not quasiprimitive on $\Delta _2$, and since *A* has index at most two in $\mathop {\textrm{Aut}}(\Gamma )$, this proves $A=\mathop {\textrm{Aut}}(\Gamma )$ and establishes part (1) of Theorem 1.1. Lemma 4.3 shows that part (2) of Theorem 1.1 holds. Finally, Lemma 4.4 shows that $\textrm{soc}(A)=\textrm{soc}(G)$ is an intransitive normal subgroup of *A*. Since the orbits of $\textrm{soc}(G)$ are preserved by *G*, Lemma 3.2 shows the orbits form the partition $\Pi$, and hence $\Gamma _Y \cong K_{1,k}$. Thus part (3) of Theorem 1.1 holds and the proof of the theorem is complete.

## Data Availability

Our manuscript has no associated data.
